# Supplementary material for: Splice-Junction-Based Mapping of Alternative Isoforms in the Human Proteome
Source: Cell Rep. Author manuscript; Available in PMC 2020 Jan 15. (PMC6961840; doi:10.1016/j.celrep.2019.11.026)
Supplement: 3 [file NIHMS1546469-supplement-3.zip › DF2/PXD0010154/Esophagus-178-P51911-YGVKPHDIFEANDLFENTNHTQVQSTLLALASMMG.pdf]

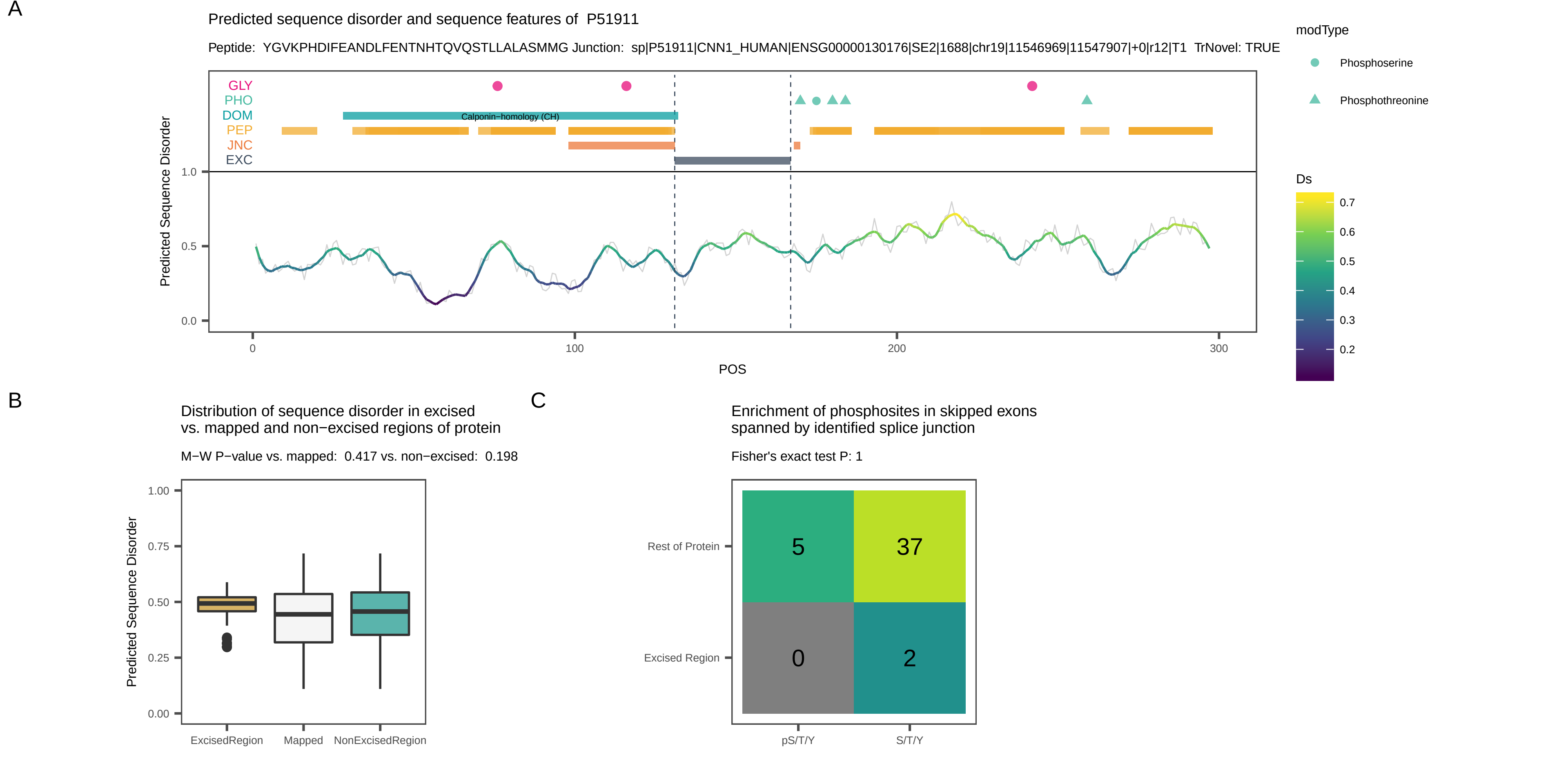
A
Predicted sequence disorder and sequence features of P51911
Peptide: YGVKPHDIFEANDLFENTNHTQVQSTLLALASMMG Junction: sp|P51911|CNN1_HUMAN|ENSG00000130176|SE2|1688|chr19|11546969|11547907|+0|r12|T1 TrNovel: TRUE
GLY
PHO
DOM
PEP
JNC
EXC
Calponin−homology (CH)
Predicted Sequence Disorder
1.0
0.5
0.0
0
100
200
300
POS
modType
Phosphoserine
Phosphothreonine
Ds
0.7
0.6
0.5
0.4
0.3
0.2
B
Distribution of sequence disorder in excised
vs. mapped and non−excised regions of protein
M−W P−value vs. mapped: 0.417 vs. non−excised: 0.198
Predicted Sequence Disorder
1.00
0.75
0.50
0.25
0.00
ExcisedRegion
Mapped
NonExcisedRegion
C
Enrichment of phosphosites in skipped exons
spanned by identified splice junction
Fisher's exact test P: 1
Rest of Protein
Excised Region
5
37
0
2
pS/T/Y
S/T/Y
